# Fer exacerbates renal fibrosis and can be targeted by miR-29c-3p

**DOI:** 10.1515/med-2021-0319

**Published:** 2021-09-13

**Authors:** Chen-Min Sun, Wen-Yi Zhang, Shu-Yan Wang, Gang Qian, Dong-Liang Pei, Guang-Ming Zhang

**Affiliations:** Department of Anesthesiology, Tongren Hospital, Shanghai Jiao Tong University School of Medicine, Shanghai 200336, China

**Keywords:** renal fibrosis, miR-29c-3p, Fer

## Abstract

**Aim:**

Renal fibrosis (RF) is a common clinical condition leading to irreversible renal function loss. Tyrosine kinase proteins and microRNAs (miRs) are associated with pathogenesis and we aim to investigate the role of Fer and its partner miR(s) in RF.

**Method:**

*In silico* reproduction of Mouse Kidney FibrOmics browser was performed to identify potential miR(s) and target gene(s). *In vivo* validation was performed in C57BL/6 mice with unilateral ureteral obstruction (UUO). *In vitro* validation was performed in rat kidney fibroblast NRK-49F cells. Mimics and inhibitors of miR-29c-3p were constructed. The target gene Fer was monitored by RT-PCR and western blotting. The levels of interleukin (IL)-6, IL-1β, and tumor necrosis factor (TNF)-α in serum and media were measured by ELISA.

**Results:**

The Fer expression and protein level were gradually increased during 14 days of UUO modeling. miR-29c-3p expression was strongly correlated with that of Fer. *In vivo* validation showed increased expressions of fibrosis-associated genes and increased phospoho-Smad3 level in the UUO model. Fer-knockdown (KD) significantly decreased expressions of fibrosis-associated genes. Pharmaceutical inhibition of Fer showed similar effects to miR-29c-3p, and miR inhibition showed a significant decrease of excretion of inflammatory factors.

**Conclusion:**

Dysregulation of miR-29c-3p and Fer plays a role in RF. Pharmaceutical or genetic inhibition of Fer may serve as the potential treatment for RF.

## 1 Introduction

Renal fibrosis (RF) is not only the main pathological basis of end-stage renal disease but also a hallmark of kidney aging [1]. Effective prevention and treatment of RF are of great significance for delaying the progression of kidney disease and reducing the occurrence of uremia. However, currently, there is no effective treatment for preventing and reversing RF [2].

MicroRNAs (miRs) are endogenous noncoding RNAs that are involved in the occurrence and development of many diseases. miR combines with different target gene mRNAs to form an RNA-induced gene silencing complex (RISC). First, the nucleus is transcribed by RNA polymerase II or III to produce pri-microRNA with a stem-loop structure. Pri-microRNA is then processed into a double strand that is composed of 70 nucleotides under the action of nuclease Drosha and its cofactor Pasha pre-microRNA. RNA–GTP and Exportin 5 deliver pre-microRNA into the cytoplasm. Next, another nuclease Dicer cuts it to produce a microRNA of 20 to 22 nucleotides in length, the double-strand microRNA, which is quickly led into RISC, where a mature single-stranded microRNA is retained in this complex. The remaining mature microRNA binds to a specific region of the 3′ untranslated end (3′UTR) of its complementary mRNA to degrade the target mRNA or inhibit its translation, thereby regulating gene expression [3].

RF is characterized by the proliferation of interstitial fibroblasts, excessive deposition of extracellular matrix (ECM), and renal tubular atrophy [4]. Transforming growth factor-β (TGF-β) is an important growth factor that activates its downstream regulatory factor Smad protein. The signaling of TGF-β is transferred from the membrane receptor to the nucleus, mediating the occurrence of renal tissue fibrosis [5]. Recent studies have found that TGF-β signaling regulates microRNA expression in renal tissue fibrosis, which features up-regulation of miR-21, miR-192, miR-491-5p, miR-382, miR-377, miR-214, and miR-433 and down-regulation of miR-29, miR-133, and the miR-200 family. It has been found in several rat models of kidney injury;microRNA changes with the severity of kidney injury, suggesting that miRs play an important role in TGF-β-mediated fibrosis [6].

The miR-29 family has been established as a protective miR in the fibrosis of several vital organs. Both miR-29a and miR-29b have been shown to play a role in RF, interacting with different gene products. Nonetheless, miR-29c has not been investigated in RF, and in the current study, we performed *in silico*, *in vitro,* and *in vivo* studies to evaluate the role of miR-29c-3p and its target gene Fer in RF.

## 2 Materials and methods

### 2.1 *In silico* analysis

Candidate miRs and genes were selected using the Mouse Kidney FibrOmics online database. The platform was a comprehensive and combined multi-omics dataset (proteomics, mRNA, and small RNA transcriptomics) of fibrotic kidneys that is searchable through the website: http://hbcreports.med.harvard.edu/fmm/. Two commonly used mouse models were utilized in the dataset, a reversible chemical-induced injury model (folic acid (FA)-induced nephropathy) and an irreversible surgically induced fibrosis model, unilateral ureteral obstruction (UUO). mRNA and small RNA sequencing, as well as 10-plex tandem mass tag (TMT) proteomics, were performed with kidney samples at different time points over the course of fibrosis development and were included in the dataset. Values were *z*-scores of the log2-normalized abundance for each molecule. The predicted interactions between miRNA and the candidate target gene were according to Targetscan DB, which was also imbedded in the platform [7].

### 2.2 Mouse models

Male BALC/c mice were obtained from Shanghai Silaike Experiment Animal Co., Ltd. All animal studies were conducted in accordance with the Ethical Guide for the Care and Use of Laboratory Animals. Mice were housed in groups of six on a 12 h light/dark cycle with access to food and water ad libitum. The experiment was commenced when mice reached 8−10 weeks of age. To construct the UUO model, mice were anesthetized with 10 μL ketamine + 1 μL xylazine/g of body weight. The abdominal cavity was opened and the left ureter was ligated twice. Sham operations were done without ureteral ligation. On day 3 and day 14, mice were sacrificed to collect renal tissues and blood samples.

### 2.3 Cell culture and transfection

Rat kidney fibroblast cell NRK-49F was obtained from Millipore Sigma and was cultured in Dulbecco's modified Eagle’s medium (DMEM) supplemented with 10% FBS. Cells were treated with TGF-β1 (10 ng/mL) for 48 h to induce fibrosis according to our pilot experience [8]. To modulate miR-29c-3p expression levels, NRK-49F cells were transfected with miR mimic or inhibitor, both synthesized by Ribobio (Guangzhou, China). The Fer inhibitor DS21360717 was obtained from ChemSrc. Adenoviral over-expression was performed using a Fer cDNA clone obtained from Origene. The Fer knockdown (KD) modeling was constructed using shRNA, the sequence of which was obtained from TRC (TRCN0000361140). The treatment dose was designated at 5 nM for the interaction for 24 h before testing. All transfections were done using Lipofectamine 2000 system according to the manufacturer’s instructions. The cells were harvested 48 h after transfection for further studies.

### 2.4 Real-time PCR

Total RNA was extracted by Trizol and converted to cDNA. Quantitative real-time PCR was carried out using SYBR ExScript RT-PCR on ABI 7500 Real-Time PCR, according to the manufacturer’s protocol. Glyceraldehyde-3-phosphate dehydrogenase (Gapdh) was used as an endogenous control. Expression of miR-29c-3p was measured using a Hairpin-it miRNA qPCR Quantitation Kit. U6 small nuclear RNA was used as an internal control for miR. The primers used are listed in Table 1.

**Table 1:** Primers used in the study (5′ to 3′)

Gene	Forward	Reverse
Acta2	CCCAACTGGGACCACATGG	TACATGCGGGGGACATTGAAG
Col1a1	GCTCCTCTTAGGGGCCACT	CCACGTCTCACCATTGGGG
Fn1	ATGTGGACCCCTCCTGATAGT	GCCCAGTGATTTCAGCAAAGG
Gapdh	AGGTCGGTGTGAACGGATTTG	GGGGTCGTTGATGGCAACA
U6	CTCGCTTCGGCAGCACA	AACGCTTCACGAATTTGCGT
	Reverse transcription: AACGCTTGACGAATTTGCGC	
miR-29c	CTAGCCTGCAGGAGGCAG	ATCCGGCCGGCCAAAAATA
	TGATAGTGAGAAAG	GTAGATAAAACAG
	Mimic: UAGCACCAUUUGAAAUCGGUUA	
	Mimic control: UUUGUACUACACAAAAGUACUG	
	Inhibitor: UAACCGAUUUCAAAUGGUGCUA	
	Inhibitor control: UUUGUACUACACAAAAGUACUG	

### 2.5 Western blotting

Protein samples (20 μg/lane) were separated by electrophoresis on 10% polyacrylamide gel and then transferred onto polyvinylidene fluoride (PVDF) membranes. The membranes were blocked with 5% nonfat milk and incubated with primary antibodies overnight at 4°C. Membranes were subsequently incubated with horseradish peroxidase (HRP)-conjugated secondary antibody at room temperature. The blots were visualized with the enhanced chemiluminescence method using an ECL kit. Primary antibodies used were Col1a1 (E8I9Z) Rabbit mAb (#91144, Cell Signaling), Actar2 Rabbit mAb (#19245, Cell Signaling), Fn1 Rabbit pAb (ab2413, Abcam), Fer Mouse mAb (Cell Signaling, #4268), and Gapdh antibody (ab9484) (Abcam).

### 2.6 Measurement of inflammatory factors

Inflammatory factors of IL-1β, IL-6, and tumor necrosis factor (TNF)-α were detected in blood samples and cell culture supernatants. BD OptEIA and ELISA kits for mouse cytokines (rat-compatible) were used, respectively.

### 2.7 Statistical analysis

*In vitro* experiments were performed in triplicates. For comparisons in more than 2 factors in more than 2 groups, the two-way analysis of variance (ANOVA), followed by *post-hoc* tests (Bonferroni) was used. For the rest of the experiments, the one-way ANOVA with Tukey’s multiple comparison test was used. The significance was designated as *p* < 0.05.

## 3 Results

**Figure 1:**
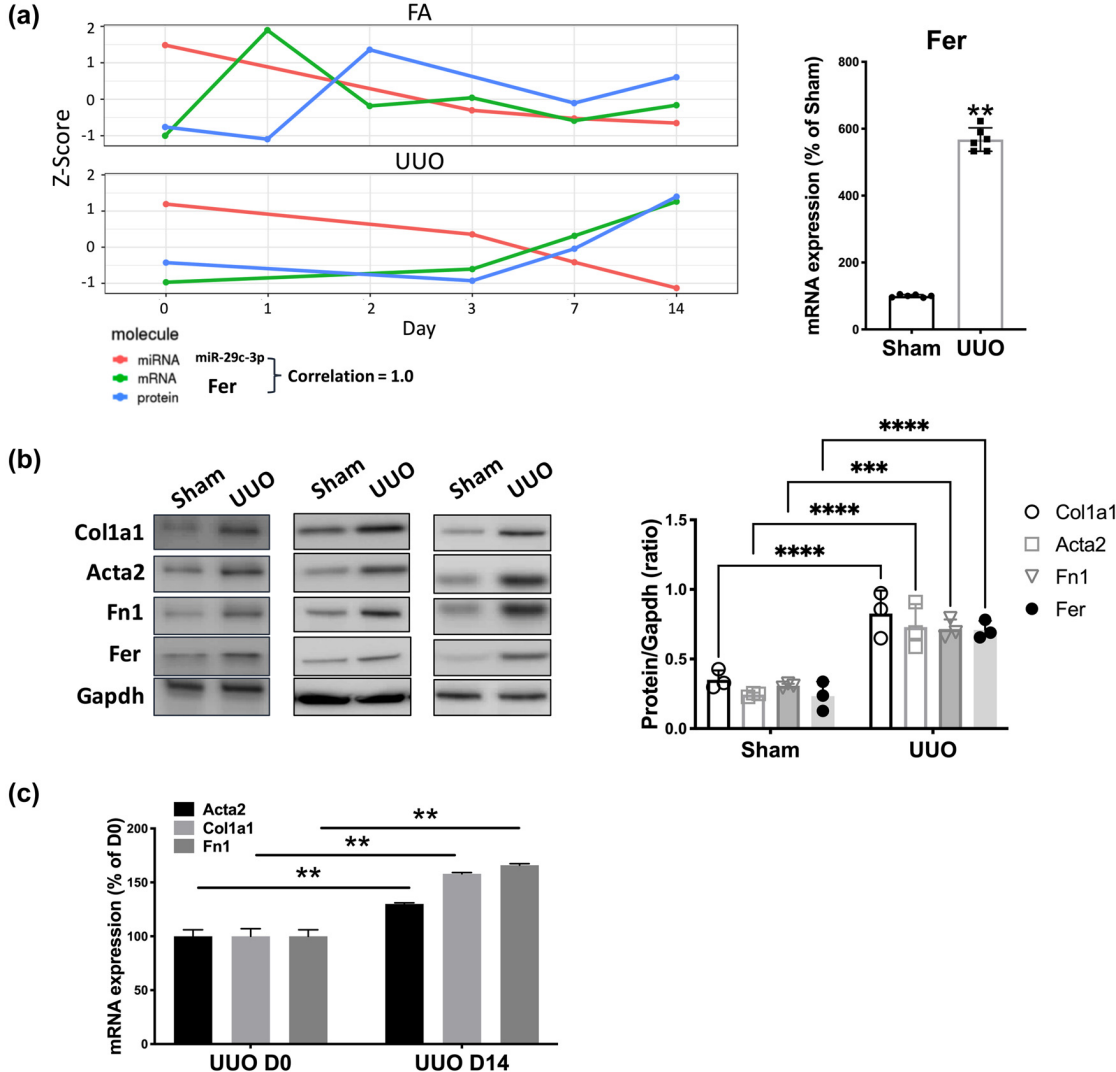
Pairs of Fer and miR-29c are dysregulated in UUO-induced RF. (a) Reproduced from the Mouse Kidney FibrOmics online database, changes in miR-29c expression and Fer expression/protein level over the 14-day course in the FA- and the unilateral ureteral obstruction (UUO)-induced RF mouse model on the left panel, and *in vivo* validation of Fer expression in kidneys harvested on day 14 post-op from mice (*N* = 6 per group) undergoing either sham surgery or UUO modeling. (b) Protein and (c) mRNA levels of fibrosis markers and Fer in kidneys harvested on day 14 post-op from mice (*N* = 6 per group) undergoing either sham surgery or UUO modeling (*P < 0.05; **P < 0.01).

Using *in silico* reproduction, we first identified continuous elevation of Fer mRNA and protein levels and the corresponding decrease of miR-29c-3p expression in the UUO model throughout 14 days of modeling (Figure 1a, left). This trend of change was not observed in the FA model. We, therefore, constructed a UUO model and detected significant elevation of Fer expression at 14 days of modeling (Figure 1a, right). Along with increased Fer level, increased levels of a series of fibrosis-associated gene products were observed in our UUO model (Figure 1b and c).

**Figure 2:**
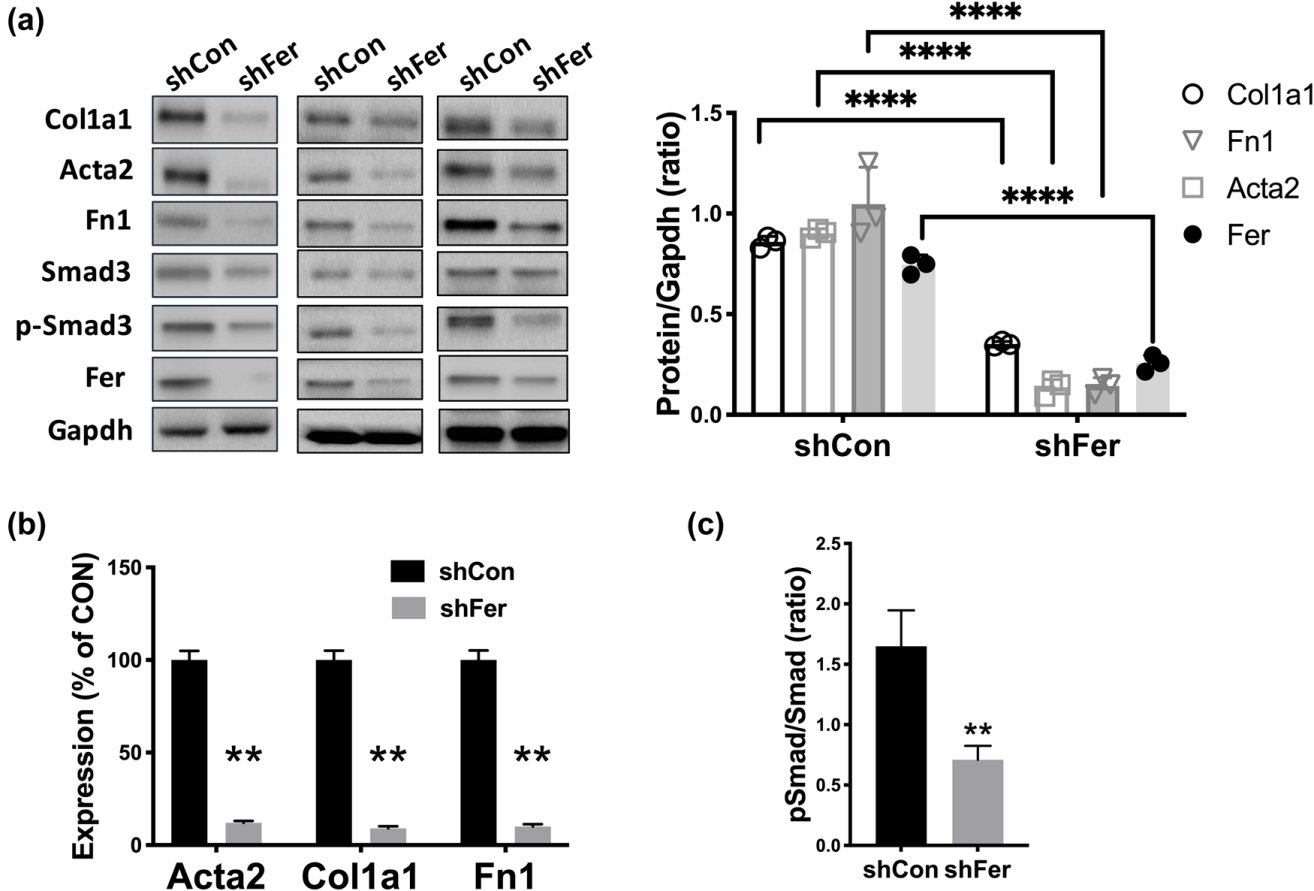
Silencing Fer ameliorates RF. Rat renal fibroblast cells NRK-49F were treated with control and shRNA targeting Fer. (a) Protein level, (b) mRNA level of fibrosis-associated genes, and (c) phospho (p)-Smad3 ratio calculated by the p-Smad3 level normalized to the total Smad3 densitometry level, all detected at 48 h after transfection (*P < 0.05; **P < 0.01).

**Figure 3:**
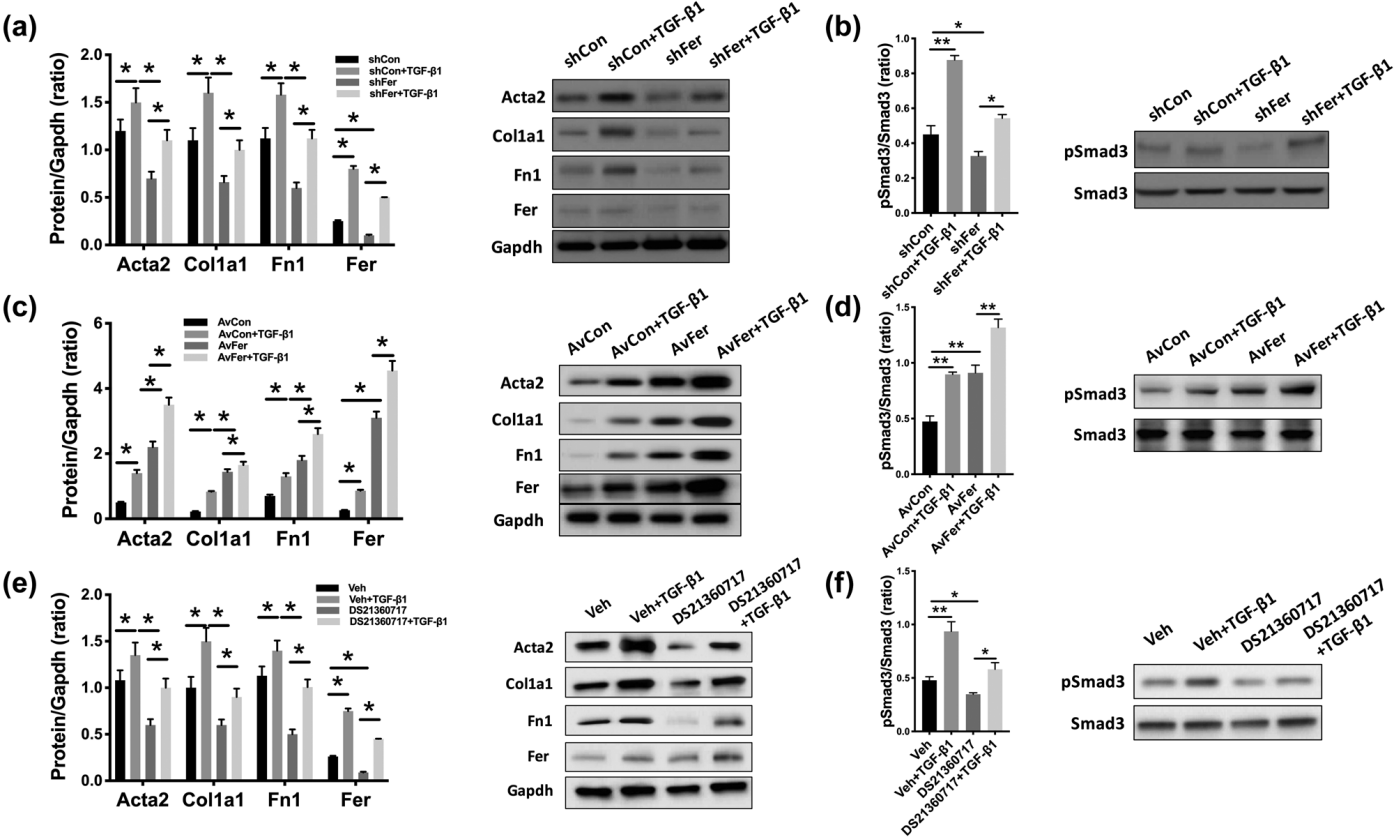
Inhibition of Fer restores TGF-ß1-induced RF. Rat renal fibroblast cells NRK-49F were modified with overexpression (adenovirus-delivered) and silencing (shRNA) of Fer and treated with TGF-β1 (10 ng/mL) for 48 h. (a) Expression of fibrosis-associated genes with TGF-ß1 addition and (b) Smad3 phosphorylation level upon Fer-KD; (c) expression of fibrosis-associated genes with TGF-ß1 addition and (d) Smad3 phosphorylation level upon Fer overexpression; (e) expression of fibrosis-associated genes with TGF-ß1 addition and (f) Smad3 phosphorylation level upon Fer inhibition (*P < 0.05; **P < 0.01).

In rat renal fibroblast cells, Fer-KD not only decreased the levels of fibrosis-associated genes at the protein (Figure 2a) and mRNA (Figure 2b) levels, respectively, but also resulted in decreased phospho-Smad3 levels (Figure 2c). When normalized to the total Smad3 level, Fer-KD also resulted in a significant decrease of the ratio (Figure 2c). We then applied TGF-ß1 treatment and found that TGF-ß1 significantly increased expressions of fibrosis-associated genes that could be abolished by Fer-KD (Figure 3a). Fer-KD also significantly reduced the phospho-Smad3 level (Figure 3b). TGF-ß1 significantly increased expressions of fibrosis-associated genes that could be further augmented by Fer overexpression (Figure 3c). Fer overexpression also significantly increased the phospho-Smad3 level (Figure 3d). Similar to Fer-KD, pharmaceutical inhibition of Fer using DS21360717 significantly abolished the expression of fibrosis-associated genes induced by TGF-ß1 (Figure 3e). DS21360717 also significantly reduced the phospho-Smad3 level (Figure 3f).

**Figure 4:**
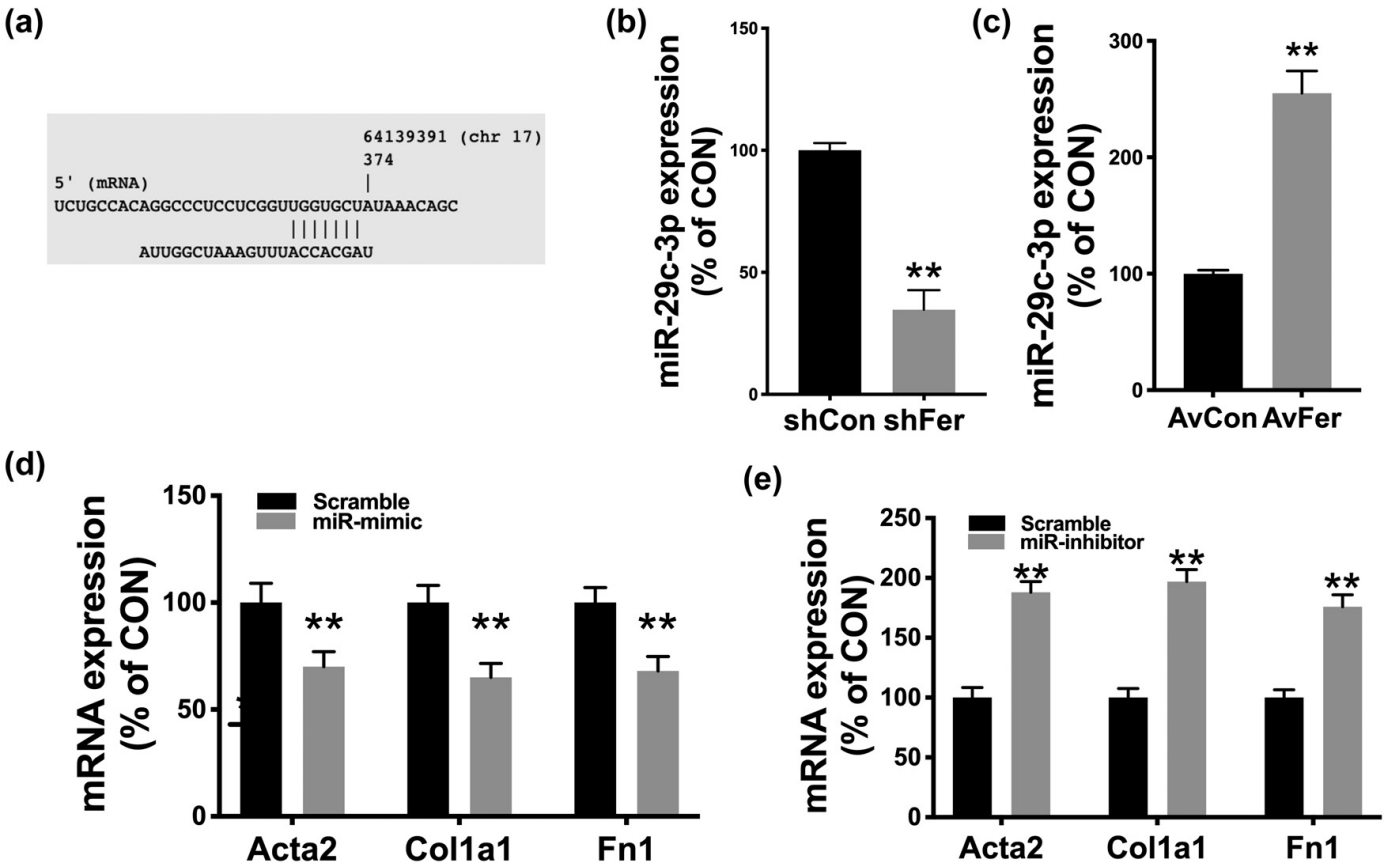
miR-29c-3p targets Fer in NRK-49F cells. (a) Binding sequence between miR-29c-3p and Fer. Expression of miR-29c-3p in cells with or without (b) Fer silencing within 48 h of shRNA transfection and (c) Fer overexpression within 48 h of adenoviral transfection. Expressions of fibrosis-associated genes in (d) miR-29c-3p mimic and (e) miR-29c-3p inhibitor-treated cells, detected 48 h post-treatment (*P < 0.05; **P < 0.01).

**Figure 5:**
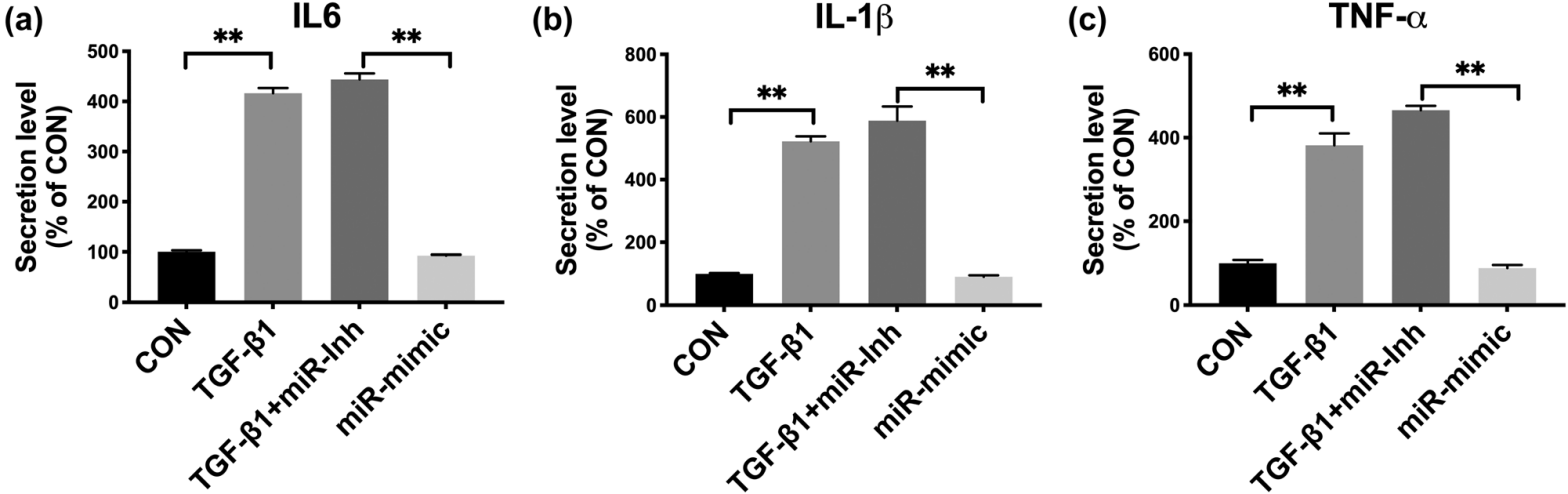
Overexpression of miR-29c-3p inhibits inflammation. (a) IL-6, (b) IL-1β, and (c) TNF-α in NRK-49F cells treated with TGF-β1 (10 ng/mL), TGF-β1 with miR-29c-3p inhibitor (miR-Inh), and miR-29c-3p mimic, all for 48 h before measurement using ELISA (*P < 0.05; **P < 0.01).

Next, we validated the target miR in the *in silico* prediction, the miR-29c-3p. We first demonstrated the matching sequence of the miR and Fer (Figure 4a). We then showed that Fer-KD resulted in the decreased miR-29c-3p expression (Figure 4b) and *vice versa* for Fer overexpression (Figure 4c), indicating dependent expressions of the pairs. miR-29c-3p mimics significantly reduced expressions of fibrosis-associated genes (Figure 4d), and the miR-29c-3p inhibitor significantly resulted in a significant increase of expressions of fibrosis-associated genes (Figure 4e). We then studied the secretion of inflammatory cytokines in NRK-49F cells. We found that TGF-ß1 induced, as expected, significantly elevated the cytokine level that could be abolished by miR-29c-3p mimics and by miR-29c-3p inhibitors (Figure 5a–c).

## 4 Discussion

In the current study, we have shown that Fer expression and protein levels were gradually increased within 14 days of UUO modeling. miR-29c-3p showed a strong correlation with Fer expression in prediction. *In vivo* validation showed increased expressions of fibrosis-associated genes and an increased phospoho-Smad3 level in the UUO model. Fer-KD significantly decreased expressions of fibrosis-associated genes. The pharmaceutical Fer inhibition and miR-29c-3p showed similar effects. The miR inhibition showed a significant decrease of excretion of inflammatory factors. Our study showed that dysregulation of miR-29c-3p and Fer plays a role in RF. Pharmaceutical or genetic inhibition of Fer may serve as the potential treatment for RF.

Fibrosis is a pathological process characterized by excessive deposition of ECM components, which is mainly associated with the synthesis and degradation of ECM components. Such imbalance transforms normal functional epithelium into nonfunctioning fibrotic tissue [9]. miRs play an extremely important role in fibrotic diseases and are fully involved in almost every step of fibrosis. miR-29 is a newly discovered miR, along its family members that are closely related to development processes in fibrosis, including heart, liver, kidneys, lung fibrosis, systemic sclerosis, etc. [10]. RF features the disappearance of renal tubular epithelial cells in the kidney tissue under the action of various pathogenic factors (inflammation, injury, hypertension, diabetes, etc.). The normal kidney tissue is replaced by inflammatory cell infiltration, activation, and proliferation of renal interstitial fibroblasts and trans-differentiation. RF promotes excessive ECM accumulation in the renal interstitial and is the main pathological basis leading to end-stage renal disease [11, 12].

In 2001, miR-29a was successfully cloned using the direct miRNA gene cloning strategy in human HeLa cells for the first time, and its analogues miR-29b and miR-29c were obtained successively [13]. They share the same seeding sequence AGCACCA with only a few nucleotides at the tail being different: miR-29a and miR-29c each containing 22 nucleotides that are only one nucleotide different, while miR-29b contains 23 nucleotides [14].

We found that the expression level of miR-29c-3p in the mouse model of renal interstitial fibrosis induced by UUO was significantly reduced, and the severity of fibrosis was closely related to the expression level of miR-29c. These results confirmed that miR-29 could be closely related to RF [15]. Fang et al. found that TGF-β1 could stimulate SMAD3 to bind to the miR-29 promoter and down-regulate the expression of miR-29. miR-29 targets the ECM and is a downstream inhibitor of fibrosis mediated by the TGF-β/Smad3 signal pathway [16]. Liu et al. found that the removal of SMAD7 promoted RF and inflammation, which were mediated by Ang-II and may be related to the enhancement of Sp1-TGF-β/SMAD3-NF-κB signaling and miR-29 expression reduction [17]. Therefore, the Smad3/miR-29 axis is likely to be an important pathway for the treatment of fibrosis. It is also reported that compared with newborn mice, the expression of miR-29 in the kidneys of adult mice was significantly up-regulated. A similar expression pattern was also observed in other organs. By establishing a Dahl salt-sensitivity (Dahl/SS) rat model of hypertension and kidney injury using a high-salt diet for more than 3 days, scholars found that miR-29b was significantly expressed in the renal medulla of SS rats using anti-sense oligonucleotides administered intravenously to knock out miR29. Multiple ECM genes (COL1a1, COL3a1, COL4a1, Col5a1, Col5a2, Col5a3, COL7A1, COL8A1, MMP2, and Itgb1) have been shown to be the target of miR-29b in 13BN rats, supporting the renal-protective role of the miR.

Our findings that Fer is targeted by miR-29c-3p in RF and that Fer inhibition could potentially ameliorate RF not only provide novel biologic insight into the miR-29 family in modulating fibrotic process but also hold promise for the development of new treatment modalities.
