# Erratum to: “Fer exacerbates renal fibrosis and can be targeted by miR-29c-3p”

**DOI:** 10.1515/med-2023-0755

**Published:** 2023-07-07

**Authors:** Chen-Min Sun, Wen-Yi Zhang, Shu-Yan Wang, Gang Qian, Dong-Liang Pei, Guang-Ming Zhang

**Affiliations:** Department of Anesthesiology, Tongren Hospital, Shanghai Jiao Tong University School of Medicine, Shanghai 200336, China

In the published article Sun CM, Zhang WY, Wang SY, Qian G, Pei DL, Zhang GM. Fer exacerbates renal fibrosis and can be targeted by miR-29c-3p. Open Med (Wars). 2021 Sep 13;16(1):1378–1385. doi: 10.1515/med-2021-0319, authors requested to replace Figures 2 and 3 due to minor errors therein during image assembling. Specifically, the second replicate (middle) of Acta2 in Figure 2a and GAPDH in Figure 3a were incorrect and the correct images were provided. The updated Figures 2 and 3 are provided. The changes do not affect the results and conclusions of the study.

**Figure 2:**
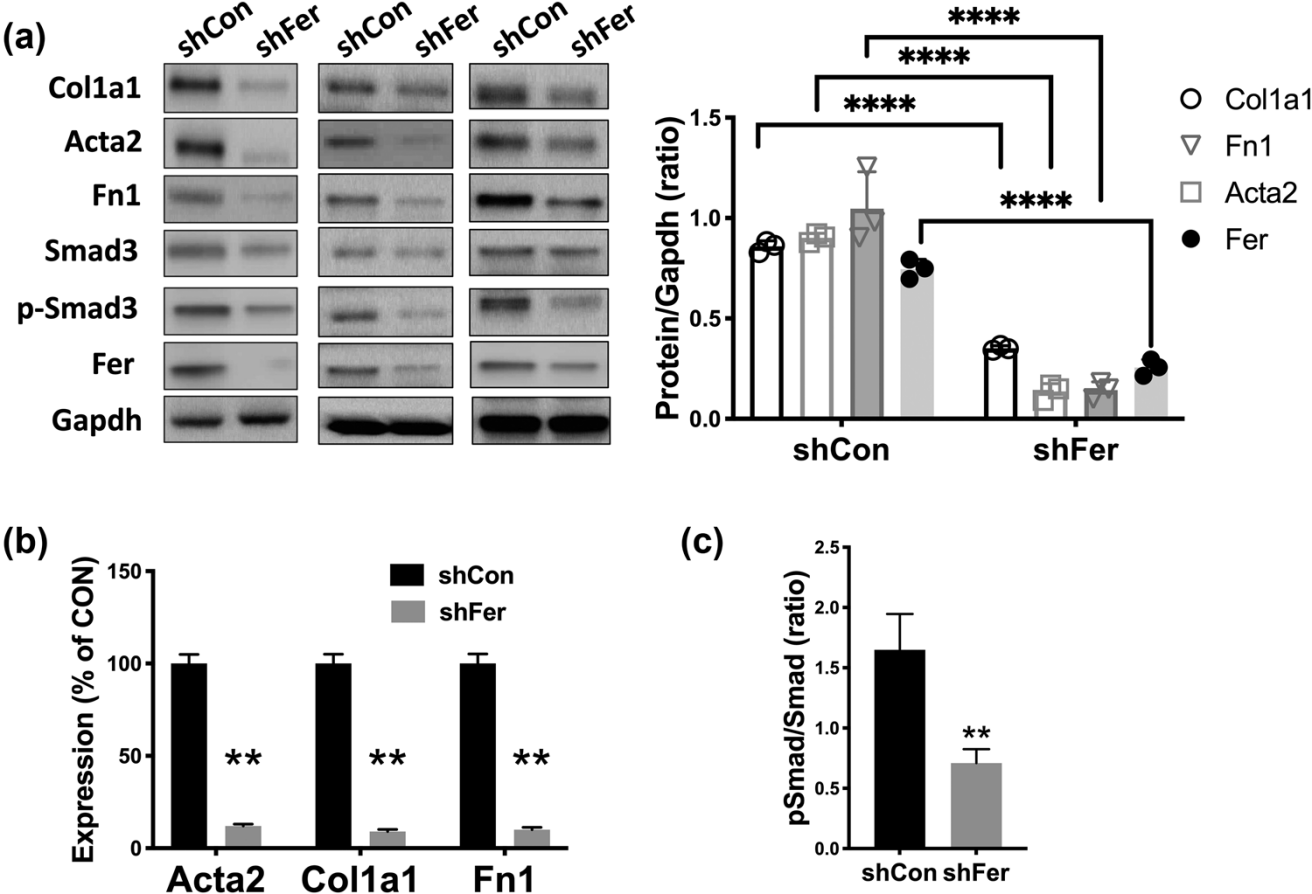
Silencing Fer ameliorates RF. Rat renal fibroblast cells NRK-49F were treated with control and shRNA targeting Fer. (a) Protein level, (b) mRNA level of fibrosis-associated genes, and (c) phospho (p)-Smad3 ratio calculated by the p-Smad3 level normalized to the total Smad3 densitometry level, all detected at 48 h after transfection (*P < 0.05; **P < 0.01).

**Figure 3:**
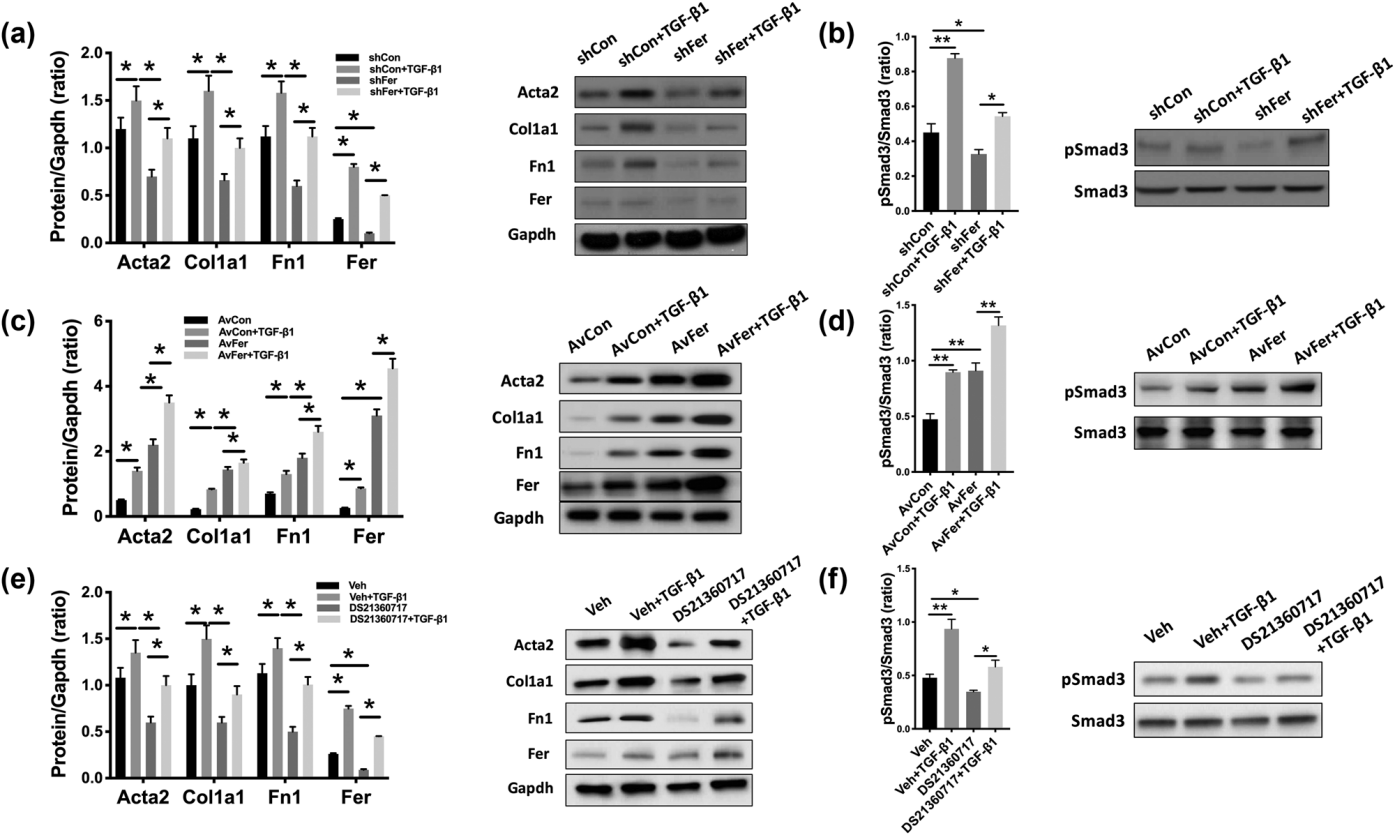
Inhibition of Fer restores TGF-ß1-induced RF. Rat renal fibroblast cells NRK-49F were modified with overexpression (adenovirus-delivered) and silencing (shRNA) of Fer and treated with TGF-β1 (10 ng/mL) for 48 h. (a) Expression of fibrosis-associated genes with TGF-ß1 addition and (b) Smad3 phosphorylation level upon Fer-KD; (c) expression of fibrosis-associated genes with TGF-ß1 addition and (d) Smad3 phosphorylation level upon Fer overexpression; (e) expression of fibrosis-associated genes with TGF-ß1 addition and (f) Smad3 phosphorylation level upon Fer inhibition (*P < 0.05; **P < 0.01).

